# Structural Insights into TIR Domain Specificity of the Bridging Adaptor Mal in TLR4 Signaling

**DOI:** 10.1371/journal.pone.0034202

**Published:** 2012-04-02

**Authors:** Zhijie Lin, Jing Lu, Weihong Zhou, Yuequan Shen

**Affiliations:** 1 State Key Laboratory of Medicinal Chemical Biology, Nankai University, Tianjin, China; 2 College of Life Sciences, Nankai University, Tianjin, China; University of Washington, United States of America

## Abstract

MyD88 adaptor-like protein (Mal) is a crucial adaptor that acts as a bridge to recruit the MyD88 molecule to activated TLR4 receptors in response to invading pathogens. The specific assembly of the Toll/interleukin-1 receptor (TIR) domains of TLR4, Mal and MyD88 is responsible for proper signal transduction in the TLR4 signaling pathway. However, the molecular mechanism for the specificity of these TIR domains remains unclear. Here, we present the crystal structure of the TIR domain of the human Mal molecule (Mal-TIR) at a resolution of 2.4 Å. Unexpectedly, Mal-TIR exhibits an extraordinarily long AB loop, but no αB helix or BB loop, distinguishing it from other TIR domains. More importantly, the Mal-TIR AB loop is capable of mediating direct binding to the TIR domains of TLR4 and MyD88 simultaneously. We also found that Mal-TIR can form a back-to-back dimer that may resemble the dimeric assembly of the entire Mal molecule. Our data demonstrate the bridge role of the Mal-TIR domain and provide important information about TIR domain specificity.

## Introduction

**Figure 1:**
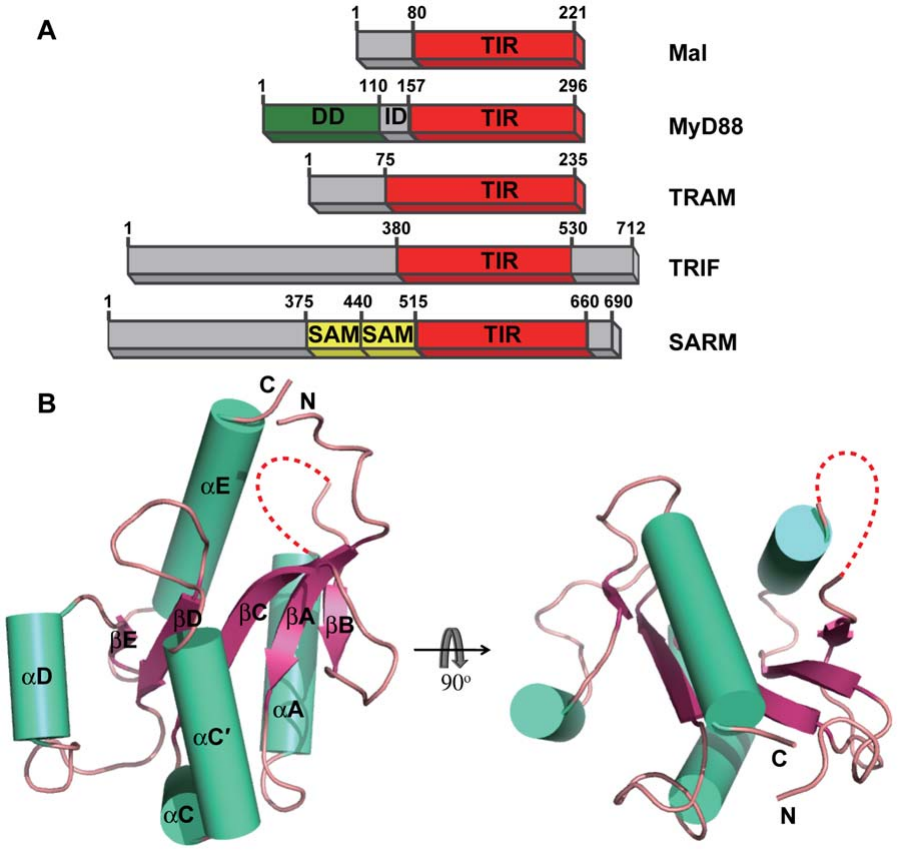
Crystal structure of Mal-TIR. (**A**) Schematic illustration of five TIR-domain-containing adaptors involved in TLR signaling. Mal: MyD88 adaptor-like protein; MyD88: myeloid differentiation factor 88; TRIF: TIR-domain-containing adaptor-inducing interferon-β; TRAM: TRIF-related adaptor molecule; SARM: sterile and HEAT/armadillo (ARM) motif protein; DD: death domain; ID: intermediate domain; SAM: sterile α-motif. (**B**) Cartoon representation of the structure of Mal-TIR. The β-strands are shown in pink, the α-helices in cyan and the connecting loops in salmon. The AB loop is colored red. The red dotted line represents the region that is not resolved in this structure.

The Toll-like receptors (TLRs) are an important group of pattern recognition receptors (PRRs) that play a critical role in host defense against pathogens throughout the animal kingdom [1], [2]. In response to various extracellular ligands, the ectodomain of a TLR dimerizes [3], [4], leading to the rearrangement of its cytoplasmic Toll/interleukin-1 receptor (TIR) domains to create a signaling platform for the recruitment of adaptor proteins. This rearrangement is followed by the expression of inflammatory and antimicrobial genes in the initiation of the adaptive immune response [5]. The specificity of TLR signaling depends on the action of different TIR domain-containing adaptor proteins (Figure 1A). Five TIR domain-containing adaptor proteins are currently known: myeloid differentiation factor 88 (MyD88), the MyD88 adaptor-like protein (Mal), TIR domain-containing adaptor-inducing interferon-β (TRIF), the TRIF-related adaptor molecule (TRAM) and the sterile and HEAT/armadillo (ARM) motif protein (SARM) [6], [7].

Mal, which is also identified as the TIR domain-containing adaptor protein (TIRAP), is essential for the TLR4 and TLR2 signaling pathways [8], [9], [10]. Mal acts as a bridge adaptor to recruit the MyD88 molecule to the activated TLR4 dimer on the plasma membrane [11]. Mal also contains several functional motifs and can be post-translationally modified. The N-terminus (residues 15–35) of the Mal molecule is a PIP2-binding domain required for membrane anchoring [12]. The C-terminus (residues 80–221) of Mal, referred to as Mal-TIR, is the TIR domain and is required for interactions with TLR4, TLR2 and MyD88 [8], [9]. Mal contains a putative TRAF6 binding motif (residues 188–196) within this TIR domain [13]. An E190A mutation in this motif results in the inhibition of TLR2- and TLR4-mediated activation of NF-κB [13]. It has also been shown that Mal can be phosphorylated by Bruton's tyrosine kinase (Btk) [14], [15] and cleaved by caspase-1 [16], and that both of these modifications are necessary for its function.

The initiation of intracellular TLR4 signaling is facilitated by the complex interactions of multiple TIR domains from TLR4, Mal and MyD88, but the mechanisms of these interactions are still controversial. Structures of isolated TIR domains from TLRs 1, 2, 10, IL-1RAPL, MyD88, the NP_177436 protein of *Arabidopsis thaliana* (*At*TIR), the TIR-like protein from *Paracoccus denitrificans* (*Pd*TLP) and the flax resistance protein L6 have been reported [17], [18], [19], [20], [21], [22], [23], but the structure of the TIR domain complex is still unknown. Several computational models have been proposed for the homomeric and heteromeric TIR-TIR interactions [22], [24], [25]. Based on the crystal structure of TLR10-TIR, a TLR4-TIR dimer model was proposed, and this model suggested that the TIR dimer interface contains residues from the BB loop, the DD loop and the αC helix [25]. However, using a decoy peptide approach and modeling, another group reported that the BB loop and the αE helix region mediate TLR4-TIR dimerization [26]. Recently, the small molecule TAK-242 (resatorvid) was found to directly bind to residue C747 in the TLR4-TIR domain and to selectively inhibit TLR4 signaling [27], [28]. This inhibitor does not affect the dimerization of TLR4 [28], but interferes with the interactions between TLR4 and Mal or TRAM [29]. Based on sequence homology, the residue C747 is located in the αC helix of the TIR domain [17].

Previous studies have identified genetic variations in TLRs and adaptor proteins associated with various infectious diseases. The most interesting single nucleotide polymorphism (SNP) is S180L in Mal. Heterozygous carriers of this variant are protected against some infectious diseases, including bacteremia, invasive pneumococcal disease, malaria and atopic dermatitis [30], [31]. In human experimental endotoxemia, individuals heterozygous for S180L produced significantly more proinflammatory cytokines in response to TLR2 and TLR4 ligands *in vivo* than those homozygous for S180L, and this response is related to an increased resistance to infection [32]. Recently, additional rare non-synonymous variants in the coding region of Mal have been found, and these variants include A9P, R13W, S55N, D96N, A100T, E132K, R143Q, R143W, S180L, R184T, E190D, V197I and X222W (a STOP codon mutated to a tryptophan codon) [31], [33], [34], [35]. Among these, D96N, E132K, R143Q and E190D have been reported to cause a loss of function in Mal [31], [33]. The D96N mutation has also been shown to impair recruitment of MyD88 to the plasma membrane and to influence the post-translational modification of Mal [33]. Thus, it appears that SNPs in Mal and other TLR-related proteins can be regarded as important genetic factors in disease. The crystal structure of Mal-TIR has been recently determined at 3.0 Å resolution and suggests that the disease-related mutants D96N and S180L may be involved in the binding interface of Mal-TIR with MyD88-TIR [36].

To elucidate the specificity of the TIR domains and the bridging role of the Mal molecule, we examined the structures of wild-type Mal-TIR at a resolution of 2.4 Å and two disease-related Mal-TIR variants, D96N and S180L. Structural analysis combined with biochemical data revealed that Mal-TIR can form a back-to-back dimer with two unexpected AB loops and bind to TLR4 and MyD88.

## Results

### Overall structure of Mal-TIR

**Figure 2:**
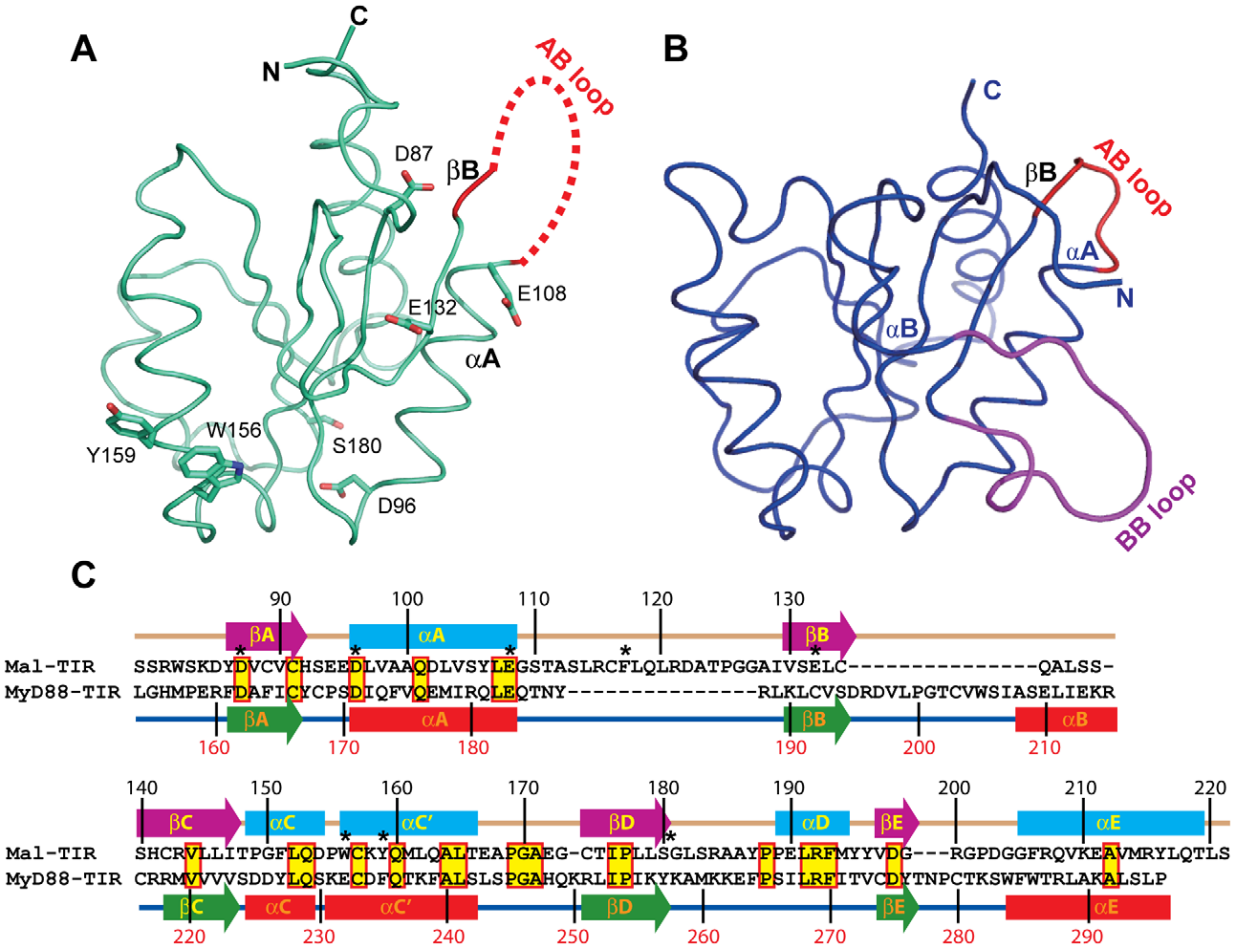
Comparison of the structures of Mal-TIR and MyD88-TIR. (**A,B**) Structural comparison of Mal (cyan) and MyD88 (blue). In Mal-TIR, the AB loop is colored red. In MyD88-TIR, the AB loop is shown in red and the BB loop in magenta. Residues D87, D96, E108, E132, W156, Y159 and S180 are shown. (**C**) Sequence alignment between human Mal-TIR and MyD88-TIR based on structural conservation. Note the differences in the region from αA to βC. Secondary structural elements of both proteins are shown as cylinders (α-helices) and arrows (β-strands). Highly conserved residues are highlighted in yellow, and residues that are similar across the group of sequences are boxed in red. Residues D87, D96, E108, F117, E132, W156, Y159 and S180 are labeled with asterisks.

The crystal structure of human Mal-TIR (residues 79–221) was determined at a resolution of 2.4 Å using the single wavelength anomalous dispersion method. The protein crystals were found to belong to the space group ***P***4_3_2_1_2 and to contain one molecule per asymmetric unit. The overall structure of Mal-TIR consists of a central five-stranded parallel sheet (βA-βE) surrounded by four helices (αA, αC-αE) on both sides (Figure 1B). As predicted, the overall topology is similar to that of the TIR domains of the receptors TLR1, TLR2, TLR10 and IL-1 RAPL and the adaptor MyD88 [17], [18], [19], [22]. Among these receptors, the TIR domain of MyD88 has the highest sequence identity (23%) to Mal-TIR. A structural comparison of the TIR domains of Mal and MyD88 yields a root-mean-square deviation (RMSD) value of 2.4 Å for 100 equivalent Cα atoms (Figure 2A). The largest differences are observed in the region from αA to βC (Figure 2A, B). In comparison with the TIR domain of MyD88, Mal-TIR has an unusually long AB loop (residues 109–129) and lacks the αB helix and the BB loop (Figure 2C). The region containing the αB helix and the BB loop has previously been recognized to mediate the interactions between different TIR domains [17], [24], [37], [38], [39], [40]. These unique features of the Mal molecule may be related to its special function as an adaptor protein by bridging the TLR2 and TLR4 signaling pathways.

### The AB loop of Mal-TIR interacts with both TLR4 and MyD88 simultaneously

**Figure 3:**
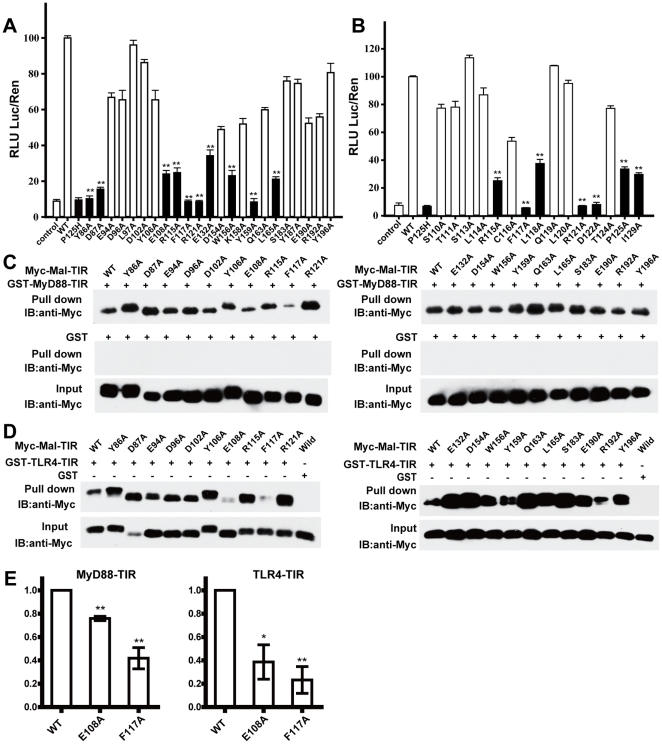
Mutational analysis of the Mal-TIR domain. (**A**) Selective mutants of solvent-exposed residues that are highly conserved across different species and used in the NF-κB reporter assay. (**B**) Alanine scanning of each residue of the AB loop (except alanine and glycine) for the NF-κB reporter assay. Black bars indicate decreased (less than 50%) NF-κB activity. RLU: relative luciferase unit; Luc: firefly luciferase activity; Ren: Renilla luciferase activity. GST pull-down assay for the MyD88-TIR and Mal-TIR variants (**C**) and for the TLR4-TIR and Mal-TIR variants (**D**). MyD88-TIR and TLR4-TIR were purified as GST fusion proteins. Mal-TIR was double-tagged with His_6_ and Myc and purified with Ni-NTA. The resulting complexes were analyzed by SDS-PAGE and Western blotting. (**E**) Bar graph of Mal-TIR wild type and mutants (E108A and F117A) binding to MyD88-TIR (left) or TLR4-TIR (right). The error bars indicate the standard error of the mean (n = 3 separate experiments). * indicates a P value<0.05, ** indicates a P value<0.001.

To screen the regions of Mal-TIR that potentially mediate interactions with TLR4 or MyD88, we performed a mutational analysis of 23 amino acid residues using a NF-κB activation dependent dual luciferase reporter assay in HEK293T cells. The selected residues are solvent-exposed and highly conserved across species (Figure S1). The Mal-TIR mutant P125H was used as the dominant negative control [9]. Among the 23 mutants, five (Y86A, D87A, F117A, R121A and Y159A) yielded the most severely (less than 20%) attenuated luciferase signals (Figure 3A). Another five mutants (E108A, R115A, E132A, W156A and L165A) exhibited modestly (greater than 20% and less than 50%) attenuated luciferase signals. Mutation of Y86A and Y159A resulted in the failure to phosphorylate the tyrosine of Mal by Bruton's tyrosine kinase, which has been found to be necessary for TLR2- and TLR4-dependent NF-κB activation [14], [15]. The other mutants, R115A, F117A and R121A, are located within the AB loop of Mal-TIR, which suggests that the AB loop may play an important role in TLR signaling. To test this hypothesis, we further mutated each residue within the AB loop to investigate each of their roles in NF-κB activation. Compared to wild type, 7 of 15 mutants (R115A, F117A, L118A, R121A, D122A, P125A and I129A) exhibited attenuated (less than 50%) NF-κB activation (Figure 3B).

Because the NF-κB activation dependent dual luciferase reporter assay is an indirect method of investigating protein-protein interactions, we used a GST pull-down assay to map regions of Mal-TIR that mediate the direct binding of Mal-TIR to MyD88-TIR or TLR4-TIR. Twenty out of the 23 myc-fused mutants were successfully expressed and purified from *E. coli* cells. In comparison to wild-type Mal-TIR, two mutants (E108A and F117A) resulted in moderately decreased binding of Mal-TIR to MyD88-TIR (Figure 3C, E). Unexpectedly, these two mutants also showed significantly reduced binding to TLR4-TIR compared to the wild-type protein (Figure 3D, E). The F117A mutation is located inside the AB loop (Figure 2C), and E108 is located at the C-terminal end of the αA helix close to the AB loop (Figure 2A). The mutation of E108 may interfere with the conformation of the AB loop. Taken together, our results suggest that the AB loop of Mal-TIR directly interacts with the TIR domains of TLR4 and MyD88. Some mutations of the Mal molecule lead to enhanced association of Mal with TLR4 or MyD88, which is consistent with previous results [15].

### Dimer packing in the Mal-TIR structure

**Figure 4:**
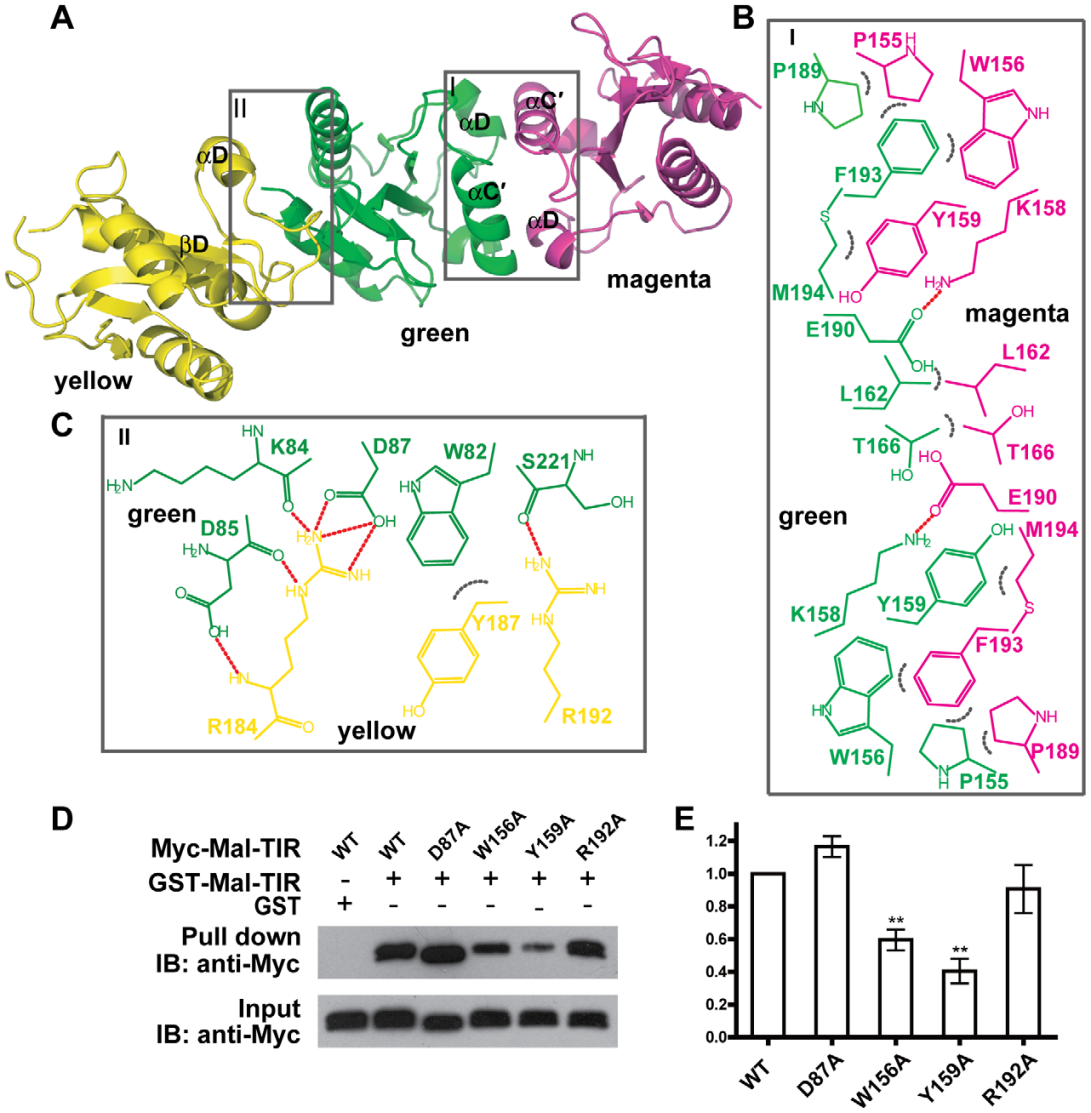
Dimeric packing of Mal-TIR. (**A**) Ribbon representation of two possible interfaces related by crystallographic packing. Chemical drawing of the key residues involved in the interactions within the interface of the symmetric dimer (**B**) and the interface of the asymmetric dimer (**C**). Hydrogen bonds are shown as dotted red lines and hydrophobic interactions as dotted gray arcs. (**D**) GST pull-down assay for the homodimerization of Mal-TIR. Wild type Mal-TIR was purified as a GST fusion protein. Double-tagged (His_6_ and Myc) Mal-TIR wild type and mutants were purified with Ni-NTA. The resulting complexes were analyzed by SDS-PAGE and Western blotting. (**E**) Bar graph of Mal-TIR homodimerization. The error bars indicate the standard error of the mean (n = 3 separate experiments). ** indicates a P value<0.001.

Several studies using the co-immunoprecipitation and yeast two-hybrid assays have reported that Mal can form homodimers through its TIR domains [9], [24], [30], [33], [34]. Based on our Mal-TIR crystal structure, two possible interfaces (green-magenta, referred to as the symmetric dimer; green-yellow, referred to as the asymmetric dimer) mediate Mal homodimerization (Figure 4A). The buried surface area, calculated by AREAIMOL [41], is 700 Å^2^ for the symmetric dimer and 500 Å^2^ for the asymmetric dimer. The interactions within the interface of the symmetric dimer primarily involve residues of the αC′ and αD helices of both monomers (Figure 4B and Figure S2A). Specifically, E190 of the green monomer forms a hydrogen bond with K158 of the magenta monomer, and P189 and F193 of the green monomer form stacking interactions with P155 and W156 of the magenta monomer, respectively. In addition to these interactions, the side chain of Y159 of the green monomer interacts hydrophobically with the side chain of M194 of the magenta monomer, and the side chains of the L162 of both monomers interact hydrophobically with each other. All of the interactions described are reciprocal.

Within the interface of the asymmetric dimer (Figure 4C and Figure S2B), R184 of the yellow monomer forms several hydrogen bonds with K84, D85 and D87 of the green monomer. W82 of the green monomer forms hydrophobic contacts with Y187 of the yellow monomer. The NH1 atom of R192 of the yellow monomer makes a hydrogen bond with the main chain carbonyl atom of S221 of the green monomer.

To validate Mal homodimerization, we mutated the residues within the interfaces of the dimers and performed GST pull-down experiments. Our results showed that the W156A and Y159A mutations located within the interface of the symmetric dimer caused a significant loss of Mal homodimer formation, while mutants D87A and R192A did not affect homodimer formation (Figure 4D, E), indicating that the symmetric dimer observed in the crystal structure is related to the Mal homodimer formed in solution.

## Discussion

Mal-TIR possesses structural features that distinguish it from other TIR domains. Previous structural studies have shown that the BB loop region of TIR is important for mediating interactions between two TIR domains [17], [24], [37], [38], [39], [40]. However, we found that the BB loop was absent in Mal-TIR. Instead of a BB loop, the Mal-TIR structure contains an extraordinarily long AB loop. Our alanine scanning experiment showed that most of the amino acid mutations within the AB loop led to impaired NF-κB activation. This result is consistent with the previous finding that the P125H mutant within the AB loop failed to immunoprecipitate with TLR4 [10]. Moreover, the mutation of another residue (E132) that is close to the AB loop also led to moderately attenuated NF-κB activation in the present study. Similarly, it has been reported that the natural variant E132K leads to a loss of function of Mal [31]. Furthermore, we found that the D87A mutation, which is located in the first βA strand of Mal-TIR near the AB loop, caused severely attenuated NF-κB signaling. In contrast, mutations at sites outside of both the AB loop and the interface of the symmetric dimer had no obvious impact on NF-κB activation. Mutations of residues distal to the AB loop also did not block the direct interactions between Mal-TIR and either MyD88-TIR or TLR4-TIR. We conclude that the AB loop of Mal-TIR plays an important role in TLR signaling through interacting with TLR4 and MyD88 simultaneously.

**Figure 5:**
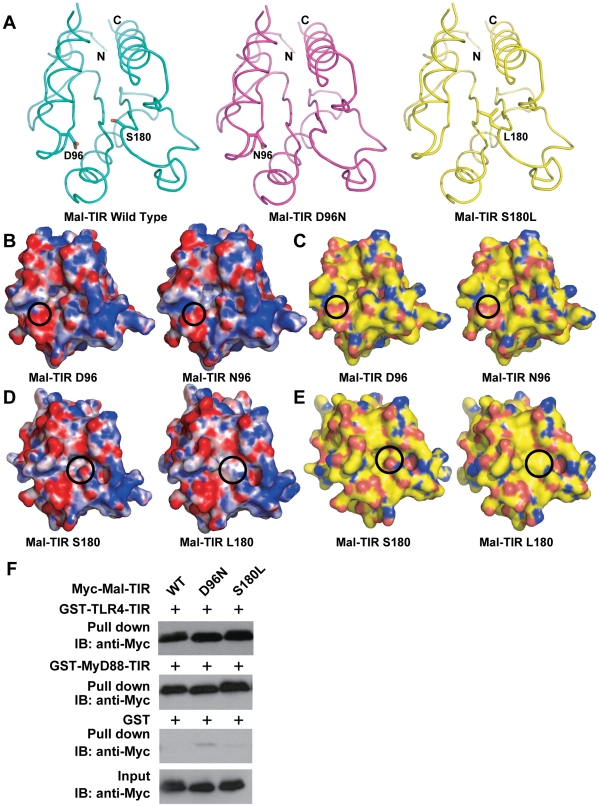
Biochemical characteristics of wild-type and mutant Mal-TIR domains. (**A**) Structural comparison between wild-type and mutant Mal-TIR domains. Crystal structures of the wild-type (cyan), D96N (magenta) and S180L (yellow) mutants of the Mal-TIR domain are shown. The residues Asp96 and Ser180 in the wild type as well as Asn96 in D96N and Leu180 in S180L are labeled. Nitrogen and oxygen atoms in the side chains are colored blue and red, respectively. (**B,D**) Electrostatic surfaces of wild type and the D96N mutant or the S180L mutant. Surfaces are colored by electrostatic potential ranging from red (−10 k_b_T/e_c_) to blue (+10 k_b_T/e_c_), where k_b_ is the Boltzmann constant, T is temperature and e_c_ is the electron charge. The electrostatic potentials were calculated by solving the Poisson-Boltzmann equation with APBS plugin of PyMol program (DeLano Scientific LLC). (**C,E**) The molecular surfaces of wild-type (left) and the D96N mutant (right) or the S180L mutant of the Mal-TIR domains. Carbon, nitrogen, oxygen and sulfur atoms are colored yellow, blue, red and orange, respectively. (**F**) GST pull-down assay for the interaction of wild-type Mal-TIR and mutants with TLR4-TIR and MyD88-TIR.

Recently, several studies have associated natural variants of Mal with various infectious diseases [30], [31], [32], [42], . To further understand the molecular mechanism underlying these associations in the Mal-TIR mutants D96N and S180L, we determined the crystal structures of these mutants. These structures showed that neither mutation interferes with the overall structure of the TIR domain (Figure 5A). We also found that the surface electronic potential map and hydrophobicity of the Mal-TIR D96N mutant does not obviously differ from that of the wild-type protein (Figure 5B, C). Our observations are inconsistent with the recent conclusion based on an *in silico* model that the D96N mutant fails to associate directly with MyD88 because this mutation affects the distribution of negative charge on the MyD88 binding site of Mal [36]. Our direct binding assay also showed that Mal-TIR D96A retains the same binding affinity to MyD88-TIR and TLR4-TIR as that observed in wild type (Figure 3), and the D96N mutant is capable of binding with MyD88-TIR (Figure 5F). Moreover, co-immunoprecipitation assays and confocal immunofluorescence microscopy have revealed that Mal D96N retains the capacity for physical interaction with MyD88 [33]. Additionally, the D96E mutant, which retains the negative charge at that position, results in the complete loss of NF-κB activation [33]. Thus, we conclude that the loss of function in Mal D96N is not caused by interference with the interaction between Mal and MyD88 but instead by another mechanism, such as altered post-translational modification of Mal [33].

The genetic variant Mal S180L has been reported to provide protection against various infectious diseases in different ethnic groups in a number of countries [30], [31], [32], [43], [44], [46]. In our crystal structure of Mal S180L, this mutation changed a negatively charged surface to a positively charged surface (Figure 5D) and a hydrophilic surface to a hydrophobic surface, thus forming a continuous hydrophobic surface (Figure 5E). This hydrophobic patch may play a role in interactions of other binding partners because the S180L mutant is capable of binding normally with MyD88-TIR and TLR4-TIR (Figure 5F).

Valkov et al. reported a similar crystal structure of the TIR domain of human Mal (accession number: 2Y92). The overall fold of Valkov's structure is highly similar to ours with a RMSD value of 0.5 Å for 121 Cα atoms (Figure S3). There are minor structural differences in the regions of some of the loops, which may be caused by different crystallization conditions and the resolution of the respective structures. The crystals of Valkov et al. were obtained in a solution containing 10–12% PEG 10,000, 5% PEG 3,350, 0.2 M NaCl, 0.1 M Tris-HCl (pH 7.3), and 20 mM DTT and diffracted to 3.0 Å resolution, while the crystals for this study were grown in *n*-cyclohexyl-2-aminoethanesulfonic acid (CHES) (pH 9.5), 150–300 mM NaCl, 10–20% glycerol and 10 mM DTT and diffracted to 2.4 Å resolution. Both structures of Mal-TIR identified the unusually long AB loop instead of the helix αB and BB loops in other known TIR domains and included two disulfide bonds (residues C89–C134 and C142–C174) and an additional DTT molecule that linked two cysteine residues, Cys91 and Cys157. In this study, we found that Mal interacts with TLR4 using the AB loop by GST pull-down assay, which was in agreement with the docking model of the TLR4-TIR and Mal-TIR complex provided by Valkov et al. [36].

However, Valkov et al. proposed that Mal-TIR binds to MyD88-TIR through an interface containing residues D96, L165 and S180. Valkov et al. also carried out a co-IP experiment to show that the D96N and L165A mutants caused significant loss of the binding of Mal-TIR and MyD88-TIR [36]. However, we performed GST pull-down assays and found that the AB loop mediated the direct binding of Mal-TIR with MyD88-TIR. We also showed that D96N and L165A can bind normally to MyD88-TIR.

Our crystal structure revealed two possible Mal-TIR dimers. The symmetric dimer is compatible with the biochemical data and most likely representative of the Mal dimer in solution. In this dimer, the AB loops of the two molecules are oriented in opposite directions, facilitating direct interaction with TLR4 and MyD88. Using a NF-κB activation report assay, we found that mutations of two residues (W156 and Y159) within the interface of the symmetric dimer yielded clearly attenuated NF-κB activation and a significant loss of Mal homodimerization (Figure 4D, E). However, the Y159F mutation, but not the Y159A mutation, apparently maintains the hydrophobic interaction with M194 (Figure 4B) and may thus maintain wild-type function. Indeed, the Y159F mutant exhibits levels of NF-κB activation comparable to wild type when Mal is overexpressed in HEK293T cells [14]. Thus, we speculate that the full-length Mal molecule forms back-to-back dimers similar to the symmetric dimer shown herein, and that the two AB loops on these Mal-TIR dimers interact with TLR4 and MyD88 simultaneously. Other mutants outside of the AB loops could also be defective in signaling, although they bind to TLR4 and MyD88 normally, which could be caused by other factors such as post-translational modification, mislocalization, etc. Further examination of structural complexes of hetero-TIR or homo-TIR domains will provide detailed information about the specific assembly of TIR domain complexes. Nevertheless, the data presented here provide important information for understanding the specificity of TIR domain assembly and the molecular basis of the bridging role of Mal in connecting TLR4 and MyD88.

## Materials and Methods

### Protein Expression and Purification

The gene-encoding residues 79–221 of human Mal protein (UniProtKB P58753) were cloned into a modified pET-32a (Novagen) vector in which the thrombin site and the S tag were replaced by the PreScission site (LEVLFQGP) and transformed into *Escherichia coli* BL21 (DE3) codon plus cells. The resulting cells were then grown in LB medium with 50 mg/L ampicillin at 25°C to A_600_ = 0.6, induced by adding isopropyl-1-thiogalactopyranoside (IPTG) to a final concentration of 200 µM and harvested after overnight. The cells were lysed in T_20_N_300_P_0.1_I_20_ buffer (20 mM Tris-HCl, pH 8.6, 0.1 mM phenylmethylsulfonyl fluoride (PMSF), 300 mM NaCl, 20 mM imidazole) using lysozyme (0.1 mg/ml) and sonication. After centrifugation at 20,000 g for 60 min, the supernatant was loaded onto Ni-NTA affinity resin (Qiagen), washed with T_20_N_300_P_0.1_I_20_ buffer and eluted with the same buffer with 300 mM imidazole. The eluted His_6_-tagged protein was digested by PreScission protease at 4°C overnight. Subsequently, the TIR domain was loaded for anion-exchange chromatography (HiTrap Q FF; GE Healthcare) in buffer A T_20_N_30_D_1_E_1_ (20 mM Tris-HCl, pH 8.6, 30 mM NaCl, 1 mM DTT, 1 mM EDTA), then eluted with gradient buffer B T_20_N_1000_D_1_E_1_ (20 mM Tris-HCl, pH 8.6, 1000 mM NaCl, 1 mM DTT, 1 mM EDTA) from 3% to 50%. The resulting sample contained protein in a buffer of 20 mM Tris-HCl, pH 8.6, 200 mM NaCl, 1 mM EDTA and 1 mM DTT. The D96N and S180L mutants were produced using standard PCR-based site-directed mutagenesis and were sequenced to confirm the mutations. The D96N and S180L mutants were expressed and purified following the same protocol used for the wild-type proteins. The selenomethionine-labeled wild-type Mal-TIR was expressed by methionine auxotroph *E. coli* B834 cells in LeMaster media and was purified following the same protocol used for the wild-type proteins.

For the GST pull-down assay, DNA fragments encoding the Myc epitope tag (EEQKLISEEDL) were inserted into a pET32a (Novagen) vector to replace the nucleotides encoding the S-tag, thioredoxin, and the thrombin-cleavable segment. The wild-type Mal-TIR was then cloned into this modified pET32a. This plasmid was used for generating 22 Mal-TIR alanine substitution variations (Y86A, D87A, E94A, D96A, D102A, Y106A, E108A, R115A, F117A, R121A, E132A, D154A, W156A, Y159A, Q163A, L165A, S183A, E190A, R192A, Y196A, D96N and S180L). All of these point mutations were created using standard PCR-based site-directed mutagenesis and confirmed by DNA sequencing. The TIR domains of the Myc-tagged wild-type and mutant Mal were purified as His_6_-fusion proteins using a Ni-NTA column. The Myc-tagged proteins were then transferred to T_20_N_200_D_1_E_1_ buffer (20 mM Tris-HCl, pH 8.6, 200 mM NaCl, 1 mM DTT, 1 mM EDTA) using a HiPrep 26/10 Desalting column (GE Healthcare).

Using PCR, the TIR domains of human Mal (79–221), MyD88 (148–296) and TLR4 (670–839) were amplified from plasmids containing full-length human MyD88 and TLR4, respectively, in the *Bam*HI and *Xho*I sites of pGEX-6P-1 (GE Healthcare). The proteins were purified on a glutathione Sepharose 4B column (GE Healthcare) and eluted with elution buffer (50 mM Tris-HCl at pH 8.0, 10 mM glutathione). The eluted GST-MyD88-TIR was transferred to T_20_K_100_D_1_E_1_ buffer (20 mM Tris-HCl pH 7.0, 100 mM KCl, 1 mM DTT, 1 mM EDTA) using a HiPrep 26/10 desalting column (GE Healthcare). The expression and purification of GST-TLR4-TIR and GST-Mal-TIR followed the same protocol as used for GST-MyD88-TIR, with the exception that the final solution contained protein in a buffer of 20 mM Tri-HCl, pH 8.0 for GST-TLR4-TIR, and 20 mM Tris-HCl, pH 8.6 for GST-Mal-TIR.

### Crystallization and Data Collection

Crystals of wild-type and Se-Met-substituted Mal-TIR were grown at room temperature with a protein concentration of 15 mg/ml. These crystals were grown for over 2 weeks using the hanging drop vapor diffusion method equilibrated against a reservoir solution of 100 mM CHES, pH 9.5, 150–300 mM NaCl, 10–20% glycerol and 10 mM DTT. The TIR domains of the D96N and S180L mutants were crystallized at 20°C for 1 month using the hanging drop vapor diffusion method with a protein concentration of 15 mg/ml and a reservoir solution containing 100 mM Tris-HCl, pH 8.0, 200–300 mM NaCl, 12–15% glycerol and 10 mM DTT. All of the crystals were frozen in a cryoprotectant consisting of the reservoir solution supplemented with 40% glycerol.

All data were collected at the Shanghai Synchrotron Radiation Facility (SSRF). Four types of crystals belong to the space group ***P***4_3_2_1_2. The wild-type Mal-TIR crystals diffracted to 2.4 Å with unit cell dimensions of ***a*** = ***b*** = 87.51 Å and ***c*** = 81.68 Å. The Se-Met-substituted crystals diffracted to 2.9 Å with unit cell dimensions of ***a*** = ***b*** = 87.92 Å and ***c*** = 82.81 Å. The crystals of D96N diffracted to 2.75 Å with unit cell dimensions of ***a*** = ***b*** = 86.92 Å and ***c*** = 81.16 Å. The crystals of S180L diffracted to 3.1 Å with unit cell dimensions of ***a*** = ***b*** = 87.17 Å and ***c*** = 80.98 Å. All of the datasets were processed and scaled using the HKL2000 software package [47].

### Structure Determination and Refinement

For the wild-type Mal-TIR, the program HKL2MAP [48] yielded three Se sites in one asymmetric unit. The initial SAD phases were calculated using PHENIX software [49]. The residues were first built automatically by the PHENIX program package and then manually using the COOT program [50] based on 2***F***_obs_ – ***F***_calc_ and ***F***_obs_ – ***F***_calc_ difference Fourier maps. The structural model was refined using the CNS program [51] and the PHENIX program [49]. The final structure had an ***R***_cryst_ value of 24.8% and an ***R***_free_ value of 26.2%.

The initial phases of the D96N and S180L mutant structures were determined by the program PHASER [52] using the wild-type structure as a template. The refinement procedures were similar to those used for the wild type. The final structure of D96N had an ***R***_cryst_ value of 24.7% and an ***R***_free_ value of 25.8%. The final structure of S180L had an ***R***_cryst_ value of 22.7% and an ***R***_free_ value of 25.4%. Detailed data collection and refinement statistics are summarized in Table 1.

**Table 1:** Data collection and refinement statistics.

Crystal name	Wild-type	Se-Met	D96N	S180L
Space group	***P***4_3_2_1_2	***P***4_3_2_1_2	***P***4_3_2_1_2	***P***4_3_2_1_2
Unit cell (Å)	***a*** = ***b*** = 87.51 ***c*** = 81.68	***a*** = ***b*** = 87.92 ***c*** = 82.81	***a*** = ***b*** = 86.92 ***c*** = 81.16	***a*** = ***b*** = 87.17 ***c*** = 80.98
Wavelength (Å)	0.9795	0.9792	0.9792	0.9789
Resolution range (Å)	50-2.4 (2.49-2.40)^a^	50-2.9 (3.0-2.9)^a^	50-2.75 (2.85-2.75)^a^	50-3.1 (3.21-3.10)^a^
No. of unique reflections	12,504	7,682	8,535	6,063
Redundancy	13.1(8.2)^a^	18.8(16.9)^a^	13.8(14.4)^a^	13.8(14.3)^a^
***R***_sym_ (%)^b^	5.9(44.0)^a^	10.5(72.3)^a^	7.8(71.7)^a^	10.7(60.5)^a^
***I***/***σ***	41.2(3.2)^a^	38.2(3.8)^a^	33(4.4)^a^	27(5.5)^a^
Completeness (%)	96.5(80.0)^a^	100(100)^a^	99.9(100)^a^	99.9(99.8)^a^
FOM		0.643		
Refinement				
Resolution range (Å)	50-2.40		50-2.75	50-3.10
***R***_crystal_ (%)^c^	24.8		24.7	22.7
***R***_free_ (%)^d^	26.2		25.8	25.4
RMSD_bond_ (Å)	0.008		0.008	0.009
RMSD_angle_(°)	1.4		1.2	1.2
Number of				
Protein atoms	971		971	973
Ligand atoms	8		8	8
Solvent atoms	11		2	2
Residues in (%)				
most favored	92.5		91.5	90.6
additional allowed	6.6		6.6	8.5
Generously allowed	0.9		1.9	0.9
disallowed	0		0	0
Average B factor (Å^2^) of Protein	61.5		69.7	70.1

a the highest resolution shell.

b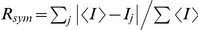.

c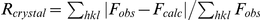.

d ***R***_free_, calculated the same as ***R***_crystal_, but from a test set containing 5% of data excluded from the refinement calculation.

### GST Pull-Down Assay

The GST fusion proteins (GST-MyD88-TIR, GST-TLR4-TIR and GST-Mal-TIR) and the Myc fusion proteins (Myc-Mal-TIR wild type and mutants) were each mixed with 30 µl glutathione Sepharose 4B beads (GE Healthcare) and incubated for binding at 4°C with rocking for 3 hours. The complexes were then washed four times with wash buffer (20 mM potassium phosphate buffer, pH 6.4, 50 mM KCl, 0.1 mM EDTA, 10 mM DTT, 0.5 mM PMSF and 0.5% Triton X-100). The bound proteins were boiled in SDS sample loading buffer, separated by SDS-PAGE (15% polyacrylamide) and analyzed using immunoblotting with anti-Myc antibodies (Sigma).

### Luciferase Reporter Assay

The full-length human Mal was used for the NF-κB luciferase reporter assay. A cDNA encoding the full-length Mal (amino acid residues 2–221) was cloned into a pCMV-Myc vector. Thirty-five alanine substitution point mutation variants (Y86A, D87A, E94A, D96A, L97A, D102A, Y106A, E108A, S110A, T111A, S113A, L114A, R115A, C116A, F117A, L118A, Q119A, L120A, R121A, D122A, T124A, P125A, I129A, E132A, D154A, W156A, K158A, Y159A, Q163A, L165A, S183A, Y187A, E190A, R192A and Y196A) along with a P125H mutant used as a negative control were generated using standard PCR-based mutagenesis and confirmed by DNA sequencing. HEK293T cells (SIBS, CAS, China) were cultured in Dulbecco's modified Eagle's medium supplemented with 10% fetal calf serum (Hyclone) and maintained at 37°C with 5% CO_2_ humidity. HEK293T cells (20,000 cells/well) in 96-well plates were transfected with an NF-κB luciferase reporter (pGL4.32; 100 ng; Promega), Renilla luciferase control reporter (pRL-TK; 50 ng; Promega) and Mal variants (pCMV-Myc; 10 ng) using Lipofectamine 2000 (Invitrogen). Following incubation for 24 hours, luciferase activity was measured using the Dual-Glo® Luciferase Assay System (Promega) according to the manufacturer's instructions. Data are expressed as the mean relative stimulation ± standard deviation (s.d.) for a minimum of three separate experiments.

### Accession Numbers

Atomic coordinates and structure factors for the reported crystal structures have been deposited in the Protein Data Bank with accession code 3UB2 for Mal-TIR, 3UB3 for D96N and 3UB4 for S180L.

## Supporting Information

Figure S1 **Functional sites of the Mal-TIR domain.** (**A**) Sequence alignment of Mal-TIR domains from different species: human (Homo sapiens, P58753), macaque (Macaca mulatta, B3Y690), chimpanzee (Pan troglodytes, B3Y685), bonobo (Pan paniscus, B3Y686), gorilla (Gorilla gorilla, B3Y687), mouse (Mus musculus, Q99JY1), domestic cow (Bos taurus, Q2LGB6), chicken (Gallus gallus, Q4U127), tropical clawed frog (Xenopus tropicalis, Q28GU9) and African clawed frog (Xenopus laevis, Q6DFE1). Accession numbers from UniProtKB are included in brackets. Secondary structural elements of human Mal-TIR are shown as cylinders (α-helices) and arrows (β-strands). Arrows indicate the residues used in functional assays. (**B**) Results of the gene reporter assays of NF-κB signaling presented on the crystal structure of the Mal-TIR. The results of the NF-κB activation assays are mapped onto the molecular surface of the Mal-TIR domain. Residues Y86, D87, E108 and E132 are located near the AB loop. Based on the luciferase assay, the residues shown to be significant (less than 20%) for Mal function deficiency are shown in red, moderately significant (greater than 20%, less than 50%) for Mal function deficiency in sky blue, and non-significant (greater than 50%) for Mal function deficiency in brown.
(TIF)

Figure S2 **Stereo view of the detailed interaction within interfaces of the symmetric dimer (A) and the asymmetric dimer (B).** Oxygen and nitrogen atoms are colored red and blue, respectively. Hydrogen bonds are shown as red dotted lines.
(TIF)

Figure S3 **Stereo view of the superimposed structures of Mal-TIR.** Our present Mal-TIR structure is colored cyan and Valkov's Mal-TIR structure (PDB code 2Y92) is colored gray.
(TIF)
