# Supplementary figures and images for: Structural Insights into TIR Domain Specificity of the Bridging Adaptor Mal in TLR4 Signaling
Source: PLoS One. 2012 Apr 2;7(4):e34202. doi: 10.1371/journal.pone.0034202 (PMC3317499; doi:10.1371/journal.pone.0034202)

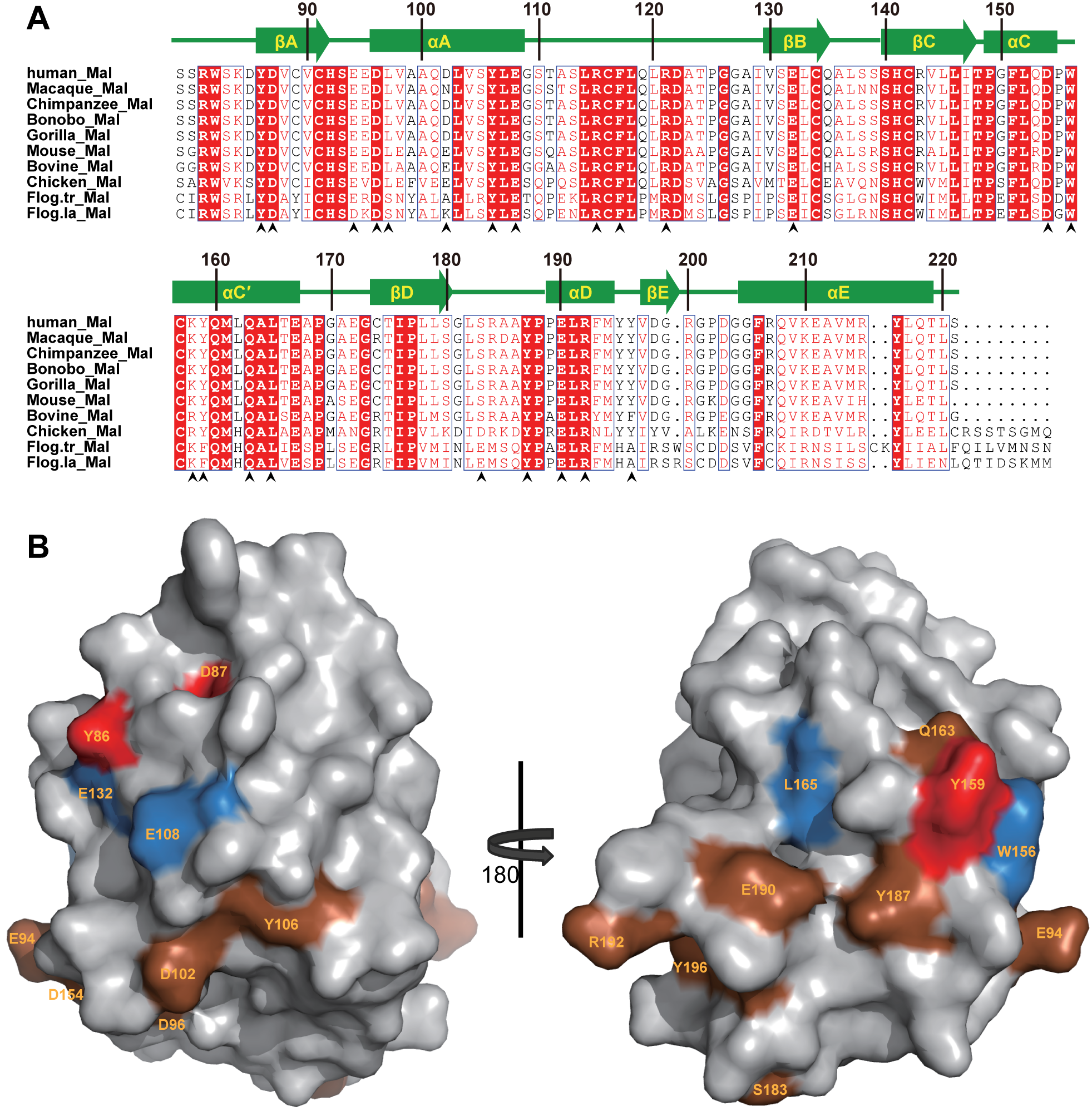

Supplement: Figure S1 — Functional sites of the Mal-TIR domain. (A) Sequence alignment of Mal-TIR domains from different species: human (Homo sapiens, P58753), macaque (Macaca mulatta, B3Y690), chimpanzee (Pan troglodytes, B3Y685), bonobo (Pan paniscus, B3Y686), gorilla (Gorilla gorilla, B3Y687), mouse (Mus musculus, Q99JY1), domestic cow (Bos taurus, Q2LGB6), chicken (Gallus gallus, Q4U127), tropical clawed frog (Xenopus tropicalis, Q28GU9) and African clawed frog (Xenopus laevis, Q6DFE1). Accession numbers from UniProtKB are included in brackets. Secondary structural elements of human Mal-TIR are shown as cylinders (α-helices) and arrows (β-strands). Arrows indicate the residues used in functional assays. (B) Results of the gene reporter assays of NF-κB signaling presented on the crystal structure of the Mal-TIR. The results of the NF-κB activation assays are mapped onto the molecular surface of the Mal-TIR domain. Residues Y86, D87, E108 and E132 are located near the AB loop. Based on the luciferase assay, the residues shown to be significant (less than 20%) for Mal function deficiency are shown in red, moderately significant (greater than 20%, less than 50%) for Mal function deficiency in sky blue, and non-significant (greater than 50%) for Mal function deficiency in brown. (TIF) [file pone.0034202.s001.tif]

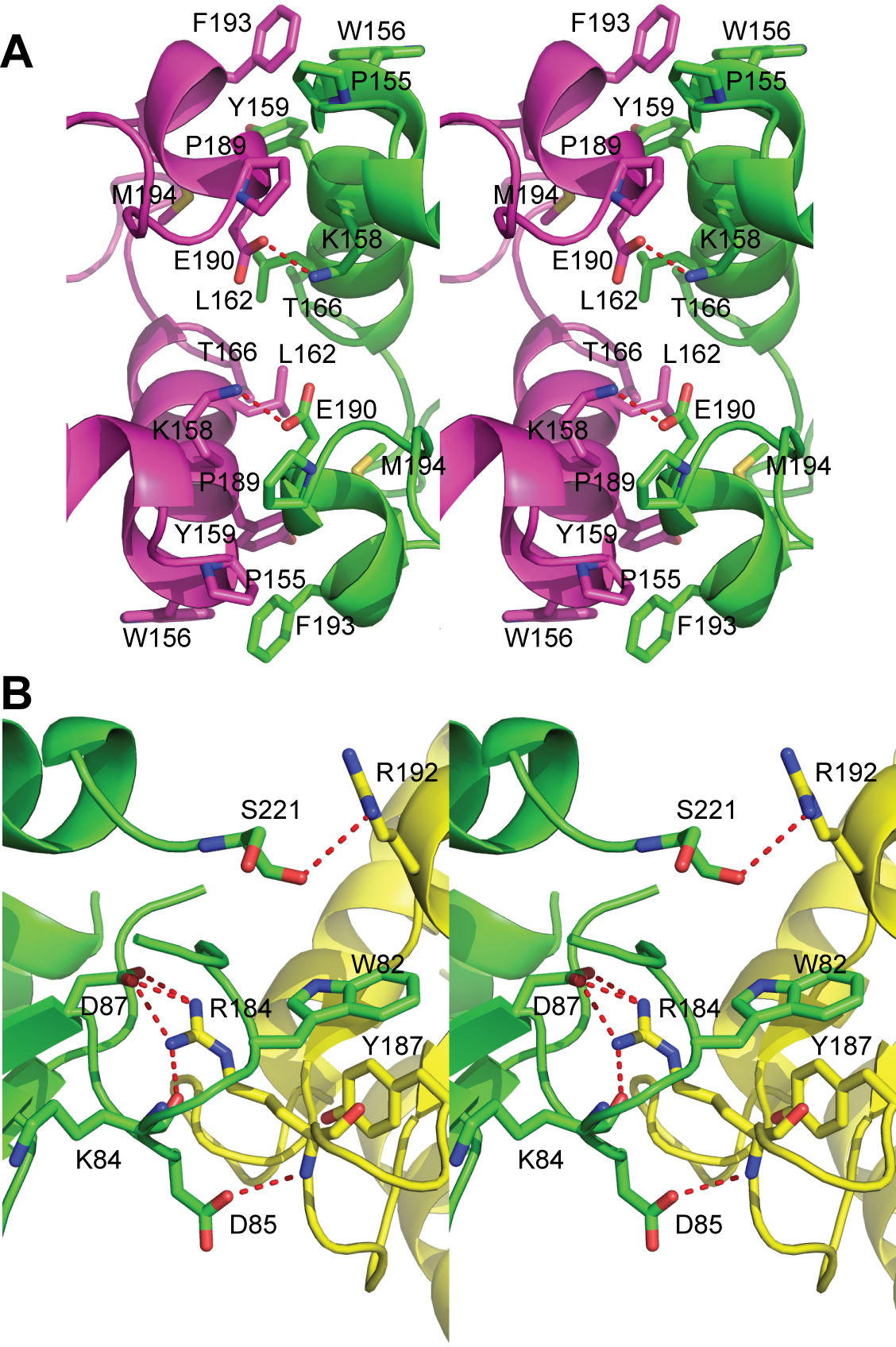

Supplement: Figure S2 — Stereo view of the detailed interaction within interfaces of the symmetric dimer (A) and the asymmetric dimer (B). Oxygen and nitrogen atoms are colored red and blue, respectively. Hydrogen bonds are shown as red dotted lines. (TIF) [file pone.0034202.s002.tif]

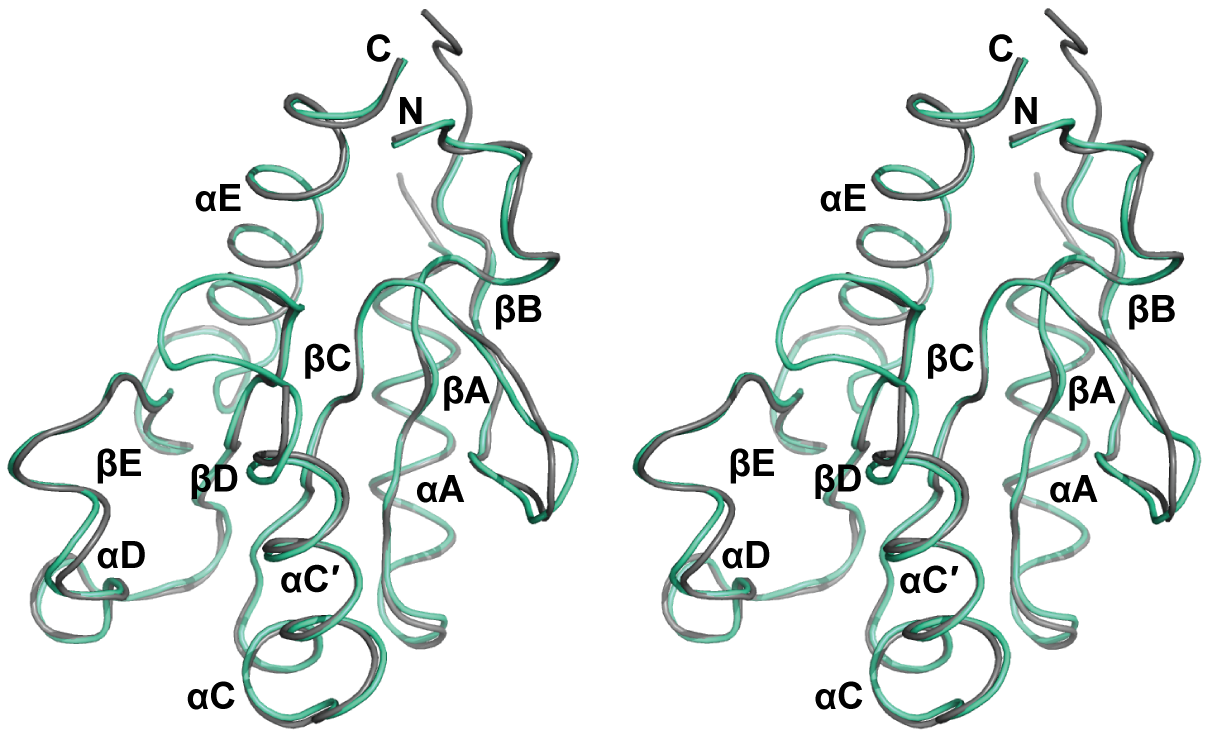

Supplement: Figure S3 — Stereo view of the superimposed structures of Mal-TIR. Our present Mal-TIR structure is colored cyan and Valkov's Mal-TIR structure (PDB code 2Y92) is colored gray. (TIF) [file pone.0034202.s003.tif]
